# Factors affecting the duration of initial medical care seeking among older rural patients diagnosed with rheumatoid arthritis: a retrospective cohort study

**DOI:** 10.1186/s41927-024-00392-9

**Published:** 2024-06-06

**Authors:** Ryuichi Ohta, Chiaki Sano

**Affiliations:** 1 Community Care, Unnan City Hospital, Unnan, 699-1221 Japan; 2 Department of Community Medicine Management, Faculty of Medicine, Shimane University, Izumo, 690-0823 Japan

**Keywords:** Rheumatoid arthritis, Rural, Older, Japan, Diagnostic window, Diagnostic delay

## Abstract

**Background:**

Early diagnosis and treatment of rheumatoid arthritis (RA) are essential to prevent progressive joint destruction and improve the quality of life (QOL) of patients. This study aimed to identify the factors associated with the duration from symptom onset to seeking initial medical care among older rural patients diagnosed with RA.

**Methods:**

This retrospective cohort study was conducted in Unnan City, Japan, using electronic patient records. Data from patients aged > 65 years, who were admitted to the Unnan City Hospital between April 2016 and March 2021, were analyzed. The primary outcome was the duration from symptom onset to the initial visit to the medical institution. Demographic factors, laboratory data, and data on symptoms were collected and analyzed using statistical tests and regression models.

**Results:**

In total, 221 participants were included in this study. The longer duration from symptom onset to medical care usage was significantly associated with age (adjusted odds ratio [OR]: 1.09, 95% confidence interval [CI]: 1.03–1.15), isolated conditions (adjusted OR: 4.45, 95% CI: 1.85–10.70), and wrist symptoms (adjusted OR: 3.22, 95% CI: 1.44–7.17). Higher education level and alcohol consumption were also associated with the duration from symptom onset to medical care usage.

**Conclusions:**

Older age, isolated conditions, and specific joint symptoms were significant factors influencing delays in seeking medical care among older rural patients with RA. Interventions to improve health literacy, increase social support, and raise awareness of RA symptoms are essential for expediting diagnosis and improving patient QOL. Further research is needed to explore additional psychosocial factors and beliefs that affect health-seeking behaviors in patients with RA.

## Background

Early diagnosis of rheumatoid arthritis (RA) is crucial for improving the quality of life (QOL) of patients. RA is prevalent in approximately 0.5% of the total population and develops mainly in middle-aged women [1]. Most patients with RA experience peripheral arthritis with a progressive clinical course [2]. Diagnosis of RA is based on classification criteria, which include clinical findings, inflammatory markers, and autoantibodies [3]. Early detection and treatment of RA are vital for preventing progressive joint destruction [4, 5]. Timely administration of disease-modifying anti-rheumatic drugs can halt or slow down the progression of RA, leading to better QOL for patients [4, 5].

Elderly-onset rheumatoid arthritis (EORA) is both prevalent and challenging to diagnose. As society ages, the incidence of RA in older patients has increased [6]. Individuals over the age of 65 years with RA present differently from those with young-onset RA (YORA) [7]. Furthermore, joint involvement patterns differ between EORA and YORA, with EORA often affecting proximal joints, such as the shoulders, neck, and femoral joints [8]. Diagnosing seronegative EORA can be particularly challenging as it requires consideration of serologic factors, such as rheumatoid factors and anti-citrullinated protein antibodies [9]. In aging societies, diagnosing EORA poses a challenge for rheumatologists, who strive to provide effective care for older patients. Previous reports have suggested several clinical approaches for diagnosing seronegative EORA, such as investigation of the persistence of peripheral arthritis and mandatory symptom follow-up [8, 9]. To avoid misdiagnosis, physicians should be aware of the diverse clinical presentations of EORA.

In addition to the varied clinical presentations of EORA, older patients face difficulties accessing medical care owing to joint symptoms and specific help-seeking behaviors (HSBs), which can delay diagnosis [10, 11]. Because of aging, older individuals tend to have joint symptoms, and thus might perceive mild symptoms as part of the natural aging process, thus leading them to endure the pain until it worsens [12, 13]. Ageism can also influence HSBs and medical care usage. Older patients may be reluctant to seek medical attention for mild symptoms, assuming that age-related symptoms are not modifiable [14, 15]. Additionally, older patients may misattribute joint pain to osteoarthritis associated with aging and may not seek medical care [16].

Furthermore, in rural areas, social factors, such as lack of medical facilities and dispersed living conditions, can further influence HSBs in older patients [10, 17]. HSBs for joint pain can significantly impact the QOL of older patients, and delays in RA diagnosis, especially in rural settings, can lead to unfavorable outcomes [18, 19]. However, no study has investigated the relationship between the duration from symptom onset to seeking initial medical care, symptom distribution, and social factors in older rural patients diagnosed with RA. Understanding the factors related to the duration of symptom onset in older rural patients with RA, including the prevalence of joint involvement and other social factors, can contribute to improving HSBs and RA care in rural settings. Therefore, this study aimed to clarify the factors influencing the duration from symptom onset to initial medical care in older rural patients with RA.

## Methods

We conducted a retrospective cohort study to investigate the factors related to the duration from the onset of initial symptoms to the initial medical care visit among older rural patients with RA in rural hospitals.

### Setting

Unnan City, located in southeastern Shimane Prefecture, Japan, is one of the most rural cities based on the previous article [20]. As of 2020, the total population of Unnan was 37,638 (18,145 men and 19,492 women), with 39% of the population aged over 65 years. The proportion of older individuals is projected to reach 50% by 2025. Unnan City is served by 16 clinics, 12 home care stations, three visiting nurse stations, and one public hospital (Unnan City Hospital). At the time of this study, the Unnan City Hospital had 281 beds, including 160 acute care beds, 43 comprehensive care beds, 30 rehabilitation beds, and 48 chronic care beds. The hospital offers specialized care in 14 medical specialties, and three rheumatology specialists provide care to all patients in the city [20]. Approximately 16 clinics, three visiting nurse stations, and 12 home care stations are functional in Unnan City. Care managers who work individually or as part of home care stations are involved in managing patient care and determining their need for professional assistance. Home care workers at the home care stations provide support to patients through physical care, assisted living, and transportation.

### Participants

We included all patients aged > 65 years, who were admitted to the Unnan City Hospital between April 1, 2016, and March 31, 2021. Data from patients who regularly visited the hospital for chronic diseases or annual health checks and were eventually diagnosed with RA were extracted for analysis. The diagnosis of RA was based on ACR (American College of Rheumatology)/EULAR (European League Against Rheu- matism) 2010 RA classification criteria. RA was diagnosed among the participants owing to bone abrasion during the initial visits.

### Primary outcome

The primary outcome was the duration between symptom onset to the initial visit to the medical institution. We determined the duration between symptom onset and diagnosis based on the data of physicians’ descriptions of the development of patients’ initial symptoms and initial visits to the hospital from the electronic patient records at the Unnan City Hospital. There was a possibility that the patients visited the local clinics, but the information could not be collected comprehensively from medical chart. As Unnan City Hospital had only one hospital providing primary and secondary care in the rural context and could diagnose and treat patients with RA, we did not include information of their previous visits to other medical institutions in this research.

### Independent variables

The background data of the participants were collected from the electronic patient records at the Unnan City Hospital. The collected patient data included age, sex, body mass index for nutritional assessment, serum creatinine level (mg/dL), estimated glomerular filtration rate (mL/min/1.73 m^2^) for renal function assessment, and Charlson comorbidity index (CCI) to assess the severity of comorbidities, including heart failure, myocardial infarction, asthma, chronic obstructive pulmonary disease, kidney disease, liver disease, diabetes mellitus, brain infarction, brain hemorrhage, hemiplegia, connective tissue diseases, dementia, and cancer [21]. Laboratory data were obtained during participants’ last visits to the hospital for chronic diseases or annual health checks. In addition, information on joint pain, including its location, number, and duration, was collected. Social factors, such as isolation and education, were assessed based on whether the participants lived with their families (living families or not) and educational backgrounds (graduation from high schools or not).

### Statistical analysis

Parametric data were analyzed using Student’s t-test, and nonparametric data were analyzed using the Mann–Whitney U test. Numerical variables were dichotomized based on the median of the following variables: duration from the appearance of initial symptoms to the hospital visit (≥ 36 days (longer duration) vs. < 36 days (shorter duration)), as the variable was not normally distributed (average: 62.6; standard deviation (SD): 91.7; median: 36; interquartile range: 727) and there was no definition of delayed usage of medical care regarding RA. We employed a univariate regression model to determine whether the time from the appearance of the initial symptoms to the hospital visit was associated with the independent variables. Based on the univariate regression model, variables significantly related to the time from the appearance of initial symptoms to hospital visits were further analyzed using a logistic regression model with a forced entry method. Regarding sample size calculation, 219 participants would be needed with 80% statistical power and 5% type 1 error to detect a difference of 10% in the duration from the appearance of initial symptoms to the hospital visit of ≥ 36 days, between the isolated and non-isolated groups, regarding one of the dependent variables of isolated conditions. Patients with missing data were excluded from the study. Statistical significance was set at *p* < 0.05. All statistical analyses were conducted using EZR (Saitama Medical Center, Jichi Medical University, Saitama, Japan), a graphical user interface for R (The R Foundation, Vienna, Austria) [22].

### Ethical considerations

The hospital ensured the anonymity and confidentiality of patient information used in this study. Information related to the study was posted on the hospital website, without disclosing any patient details. The hospital representative’s contact information was provided on the hospital website to address any questions regarding the study. All participants were informed of the purpose of the study, and they provided informed consent. This study was approved by the Clinical Ethics Committee of our institution (approval code: 20,220,007).

## Results

### Participants’ selection process

**Fig. 1:**
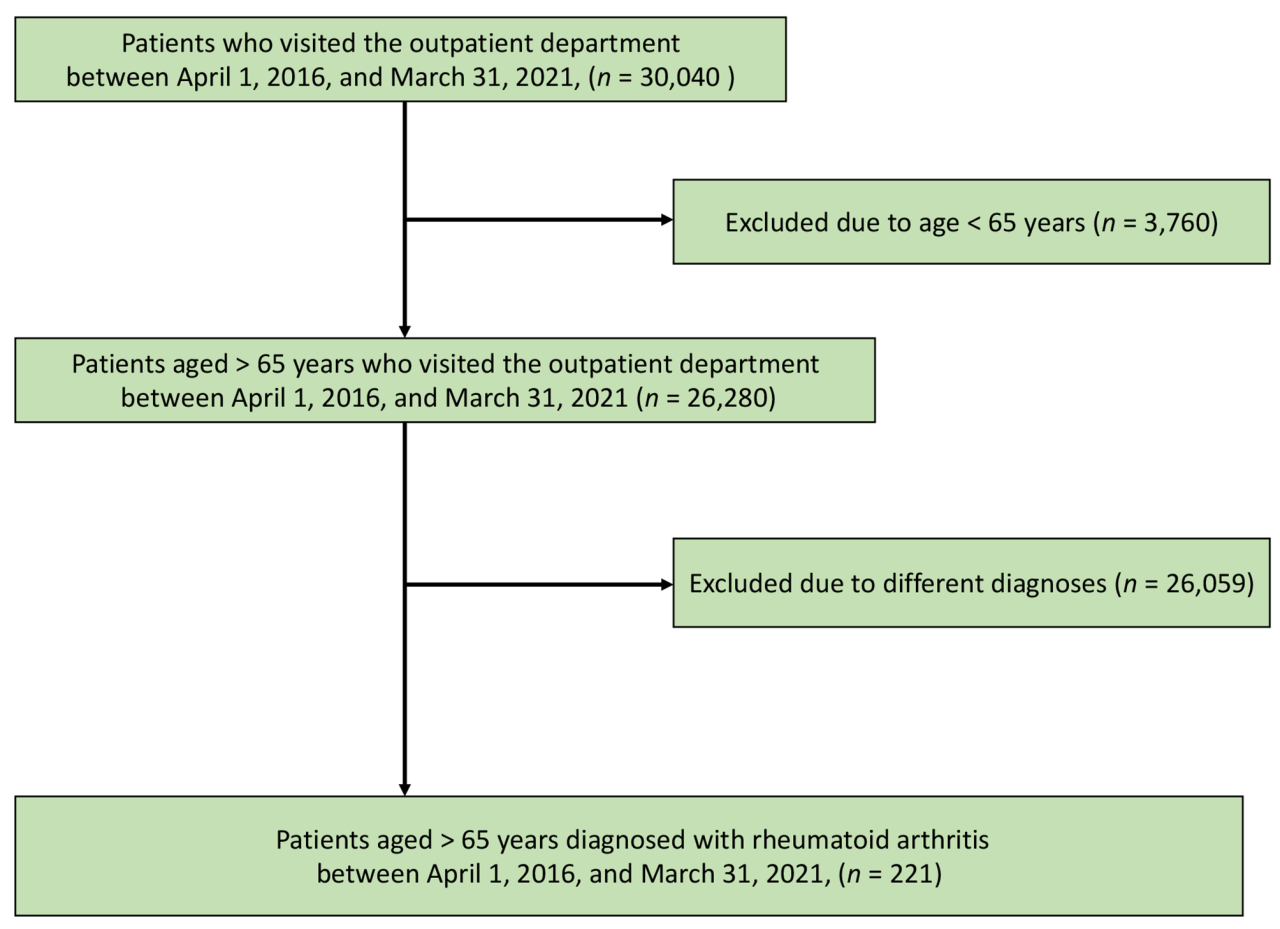
Participant selection flowchart

Of the 30,040 patients who visited the Unnan City Hospital during the study period, 26,280 were aged > 65 years. After excluding 26,059 patients with other diagnoses and those without regular hospital visits, 221 patients were evaluated. A patient inclusion flowchart is presented in Fig. 1.

### Demographic characteristics of the participants

In this study, we analyzed 221 participants to investigate the differences in various factors and symptoms between the two groups, based on the duration from the appearance of initial symptoms to the hospital visit. Of the total participants, 113 had a longer duration and 108 had a shorter duration. Participants with a longer duration had a mean age of 84.92 years (SD = 8.57), whereas those with a shorter duration had a mean age of 76.51 years (SD = 12.22) (*p* < 0.001). The male population accounted for 28.1% of the total sample, with a higher percentage of men (34.3%) in the group with a shorter duration than in the group with a longer duration (22.1%) (*p* = 0.052).

No significant differences were observed in weight, height, body mass index, and several blood markers (hemoglobin, serum albumin, estimated glomerular filtration rate, creatinine, white blood cell count, and lactate dehydrogenase levels) between the two groups. However, significant differences were observed in alcohol consumption and higher education level between the two groups (*p* = 0.002 and *p* < 0.001, respectively). Smoking, isolated living conditions, and the number of medications were not significantly different between the groups. The median CCI score was 4.00, with a slightly varied distribution (*p* = 0.041). Significant differences were observed in hand pain (*p* = 0.008), hip pain (*p* = 0.009), and wrist pain (*p* = 0.001) between the two groups. Other symptoms, such as ankle pain, fever, neck pain, and systemic pain, showed potential trends toward significance; however, most other symptoms did not significantly differ between the two groups. The majority (78.3%) of the participants reported bilateral symptoms, and no significant difference was observed in the laterality distribution, C-reactive protein, erythrocyte sedimentation rate, anti-citrullinated protein antibodies, and rheumatoid factors between the two groups (Table 1).

**Table 1:** Demographic data of participants

Factor	Total	Longer duration	Shorter duration	*P* value
n	221	113	108	
Age, mean (SD)	80.81 (11.31)	84.92 (8.57)	76.51 (12.22)	< 0.001
Male sex (%)	62 (28.1)	25 (22.1)	37 (34.3)	0.052
Weight, mean (SD)	49.57 (11.40)	49.48 (12.02)	49.66 (10.75)	0.91
Height, mean (SD)	152.70 (9.79)	152.20 (9.60)	153.22 (10.00)	0.44
BMI, mean (SD)	21.18 (4.03)	21.24 (4.31)	21.12 (3.73)	0.828
Alcohol (%)	79 (35.7)	29 (25.7)	50 (46.3)	0.002
Smoking (%)	28 (12.7)	13 (11.5)	15 (13.9)	0.687
Isolated condition (%)	43 (19.5)	34 (30.1)	9 (8.3)	< 0.001
Higher education (%)	128 (57.9)	45 (39.8)	83 (76.9)	< 0.001
Medicine number, mean (SD)	6.38 (3.95)	5.94 (3.92)	6.83 (3.96)	0.093
CCI, median (IQR)	4.00 [1.00, 7.00]	4.00 [2.00, 7.00]	4.00 [1.00, 7.00]	0.041
Hemoglobin, mean (SD)	11.58 (1.73)	11.66 (1.66)	11.50 (1.81)	0.486
Serum albumin, mean (SD)	3.32 (0.65)	3.37 (0.68)	3.27 (0.61)	0.261
eGFR, mean (SD)	70.33 (20.61)	71.02 (21.36)	69.62 (19.88)	0.616
Creatinine, mean (SD)	0.75 (0.46)	0.74 (0.55)	0.76 (0.36)	0.758
WBC, mean (SD)	6310 (197)	6390 (203)	6240 (192)	0.574
LDH, mean (SD)	197.38 (45.01)	196.12 (40.67)	198.69 (49.30)	0.671
CRP, mean (SD)	4.90 (8.36)	4.49 (10.53)	5.33 (5.16)	0.458
ESR, mean (SD)	61.17 (30.47)	58.04 (30.50)	64.51 (30.22)	0.114
RF, mean (SD)	92.69 (271.14)	76.43 (173.49)	110.03 (346.28)	0.358
ACPA, mean (SD)	61.81 (150.54)	62.06 (139.11)	61.54 (162.49)	0.98
Symptoms				
Ankle (%)	11 (5.0)	9 (8.0)	2 (1.9)	0.06
Back (%)	23 (10.4)	11 (9.7)	12 (11.1)	0.827
Edema (%)	13 (5.9)	8 (7.1)	5 (4.6)	0.571
Elbow (%)	22 (10.0)	15 (13.3)	7 (6.5)	0.116
Fatigue (%)	25 (11.3)	13 (11.5)	12 (11.1)	1
Fever (%)	31 (14.0)	11 (9.7)	20 (18.5)	0.08
Foot (%)	23 (10.4)	13 (11.5)	10 (9.3)	0.662
Hand (%)	62 (28.1)	41 (36.3)	21 (19.4)	0.008
Hip (%)	41 (18.6)	13 (11.5)	28 (25.9)	0.009
Knee (%)	33 (14.9)	20 (17.7)	13 (12.0)	0.262
Neck (%)	29 (13.1)	10 (8.8)	19 (17.6)	0.072
Shoulder (%)	69 (31.2)	31 (27.4)	38 (35.2)	0.246
Stiffness (%)	23 (10.4)	15 (13.3)	8 (7.4)	0.188
Systemic pain (%)	27 (12.2)	9 (8.0)	18 (16.7)	0.064
Wrist (%)	56 (25.3)	40 (35.4)	16 (14.8)	0.001
Laterality (%)				1
Bilateral	173 (78.3)	88 (77.9)	85 (78.7)	
Right	33 (14.9)	17 (15.0)	16 (14.8)	
Left	15 (6.8)	8 (7.1)	7 (6.5)	

ACPA, anti-citrullinated protein antibodies; BMI, body mass index; CCI, Charlson comorbidity index; CRP, C-reactive protein; Duration, the duration between symptom onset to the initial visit to the medical institution; eGFR, estimated glomerular filtration rate; ESR, erythrocyte sedimentation rate; LDH, lactate dehydrogenase; RF, Rheumatoid factors; SD, standard deviation; WBC, white blood cell; IQR, interquartile range

### Relationship among the appearance of the initial symptoms, hospital visits, and demographic factors

To further analyze the associations between various factors that were significant in the univariate regression, we performed a logistic regression analysis with the duration from the appearance of initial symptoms to the hospital visit as the outcome variable. A 1-year increase in age was significantly associated with a 9% increase in the odds of the outcome (adjusted odds ratio [OR] 1.09; 95% confidence interval [CI]: 1.03–1.15, *p* = 0.0019). Individuals with an isolated condition had 4.45 times higher odds of the outcome compared with that of those without an isolated condition (95% CI: 1.85–10.70, *p* = 0.00089). Similarly, individuals with wrist symptoms had 3.22 times higher odds of outcome of a longer duration than that of those without such symptoms (95% CI: 1.44–7.17, *p* = 0.0043). However, other factors, such as male sex, alcohol consumption, higher education, higher CCI, and specific symptoms (ankle, hand, and hip pain) did not show significant associations with the outcome (Table 2).

**Table 2:** Results of logistic regression for longer duration between symptom onset to initial visit to the medical institution

Factor	Adjusted OR	95% CI	*P* value
Age	1.09	1.03–1.15	0.0019
Male sex	0.77	0.37–1.59	0.48
Alcohol consumption	0.77	0.35–1.72	0.52
Higher education	0.5	0.22–1.14	0.099
Isolated condition	4.45	1.85–10.70	0.00089
CCI	0.73	0.48–1.12	0.15
Ankle symptoms	2.73	0.44–16.90	0.28
Hand symptoms	1.68	0.78–3.62	0.18
Wrist symptoms	3.22	1.44–7.17	0.0043
Hip symptoms	0.51	0.22–1.20	0.12

CCI, Charlson comorbidity index; OR, odds ratio; CI, confidence interval

## Discussion

Our study aimed to identify the factors associated with the duration from symptom onset to the initial medical care visit among older patients diagnosed with RA in rural Japan. This investigation is particularly relevant because of the increasing older population in Japan and its implications for healthcare and social systems. Our comprehensive analysis reveals several determinants, such as age, isolated conditions, higher education, and particular joint pains that significantly influence the delay in seeking medical care.

Firstly, age appears to be a significant factor for the timing of medical usage, and those in the group with a longer duration from symptom onset to medical care were significantly older than their counterparts in the shorter duration category. This finding suggests that older individuals tend to perceive joint pain as a natural aspect of aging, leading to a delay in seeking medical advice. Another plausible interpretation is that the social and psychological barriers faced by older individuals are potentially shaped by ageism, as previously discussed [15]. Additionally, we observed a higher representation of men in the group that sought medical attention in a shorter duration than in a longer-duration group. Although the reason remains unclear, it might be related to sex-specific perceptions of pain, health-seeking behaviors, or manifestations of RA symptoms in men [23, 24]. The age discrepancy observed in our study is consistent with those reported in previous studies, suggesting that older age is a determinant of delayed RA diagnosis [25, 26]. The longer delay in seeking medical care in patients aged > 84 years suggests that older patients may either underestimate their symptoms or attribute them to the normal aging processes [27]. This observation is consistent with those of previous studies, indicating that older adults are more likely to attribute joint pain and related symptoms to the natural course of aging rather than to a specific pathology, such as RA [20, 28].

Isolated conditions were another primary focus of our study as determinants of delays in RA care. Our findings indicate that older patients with RA living in isolation, tend to seek medical care with delayed timing compared with those living with families and relatives. A previous report in a rural setting supports this result and shows that isolated patients face challenges in accessing medical care owing to a lack of resources [29]. Furthermore, another study suggested that isolated conditions may decrease health literacy, leading to fewer opportunities for older patients to obtain information regarding their joint pain and assess the severity of symptoms based on the timing of medical care usage [30]. Older rural patients in isolation frequently misinterpret isolated RA symptoms without mutual support from relatives and families, considering them to be the results of mere aging or physical labor [31]. Rural settings have previously had robust social connections and mutual support for each other’s conditions [32, 33]. However, as aging societies progress, younger generations leave rural areas, leading to a decline in social support for older generations [34]. These results indicate that the present rural conditions, characterized by isolation and less mutual support, affect RA care in rural settings, necessitating interventions to improve healthcare. Based on this study and a previous report, local governments should intervene and enhance social support for isolated individuals to facilitate the early detection of joint pain with the possibility of RA.

Alcohol consumption and higher education levels exhibited notable differences between the two groups, suggesting potential behavioral and socioeconomic factors that might influence the timing of medical consultations. Individuals with higher educational backgrounds may have better access to healthcare information and the means to act on it [35, 36]. However, the relationship between alcohol consumption and RA onset to consultation time requires further investigation. One possible interpretation is that those who consume alcohol might be more socially active and, therefore, more motivated to seek treatment early because of the impact of RA symptoms on their social lives [37]. However, this hypothesis remains speculative and requires additional investigation [38]. Our results also established a significant link between alcohol consumption and higher education level and the duration from symptom onset to hospital visit. Patients with higher educational levels are more likely to seek medical advice. This finding is consistent with prior research findings, indicating that education can influence HSBs, possibly owing to better health literacy or greater awareness of the disease [39, 40].

Furthermore, we observed significant differences in the symptoms between the two groups. Hand, hip, and wrist pain were pivotal symptoms influencing HSBs. The prominence of these areas might be attributed to their essential role in daily activities, making their impairment more noticeable and impactful, thus prompting a quicker response [41]. The significant differences in hand, hip, and wrist pain echo the findings of earlier studies that stressed the importance of these symptoms in prompting patients to seek medical attention [42]. Moreover, symptoms, such as ankle pain, fever, and neck pain, showed potential trends, suggesting a more extensive list of symptoms that might influence HSBs, including polymyalgia rheumatica and giant cell arthritis [43]. This observation expands the existing literature by emphasizing the importance of a wide range of symptoms rather than only a few “classic” RA symptoms.

Despite valuable insights gained from this study, we acknowledge some limitations. First, being a retrospective analysis, our results are susceptible to recall bias as patients may not accurately remember the timing or intensity of their initial symptoms. Second, our study focused on rural patients in a specific region of Japan, making the generalization of these findings to other geographical or cultural settings challenging. Additionally, our study did not consider the role of primary healthcare providers in RA diagnosis, as knowledge and awareness of RA can also lead to delays in RA diagnosis. Furthermore, psychosocial factors and personal beliefs about health, which could provide deeper insights into delayed HSBs, were not examined in this study.

## Conclusions

Our study underscores the multifaceted determinants that influence the delay from symptom onset to seeking medical care in older rural patients with RA. Factors such as age, isolation, higher education, and specific joint pain play pivotal roles in the duration of seeking medical care. By elucidating these factors, healthcare providers and policymakers can design targeted interventions to expedite RA diagnosis, particularly in rural settings. It is imperative to enhance public awareness regarding the symptoms of RA and the benefits of early diagnosis, particularly among older adults. Given the limitations of this study, future research should consider broader demographics and delve deeper into the individual, social, and systemic factors that affect early diagnosis of RA. Our findings offer a foundation for developing strategies to improve the timeliness of RA diagnosis, which can significantly affect a patient’s QOL. Further research is needed to delve deeper into the mechanisms behind these associations, particularly the role of socioeconomic factors and health behaviors, in the context of RA care-seeking.

## Data Availability

All relevant datasets used in this study are presented in the manuscript.

## Abbreviations

RA: Rheumatoid arthritis
QOL: Quality of life
EORA: Elderly-onset rheumatoid arthritis
YORA: Young-onset rheumatoid arthritis
HSBs: Help-seeking behaviors
CCI: Charlson comorbidity index
OR: Odds ratio
CI: Confidence interval
